# MALAT1 is involved in type I IFNs-mediated systemic lupus erythematosus by up-regulating OAS2, OAS3, and OASL

**DOI:** 10.1590/1414-431X20209292

**Published:** 2020-04-17

**Authors:** Fei Gao, Yuan Tan, Hong Luo

**Affiliations:** Dermatology Department, The First Hospital of Changsha City, Kaifu District, Changsha, China

**Keywords:** MALAT1, OAS2, OAS3, OASL, Systemic lupus erythematosus, Type I IFNs

## Abstract

Systemic lupus erythematosus (SLE) is an autoimmune disease associated with an aberrant activation of immune cells partly due to the dysfunction of cytokines such as type I interferons (IFNs). Long non-coding RNA MALAT1 has been found to play a pathogenic role in SLE; however, the underlying mechanisms are still poorly understood. Bioinformatics analysis showed the up-regulation of type I IFN downstream effectors OAS2, OAS3, and OASL (OAS-like) in CD4^+^ T cells, CD19^+^ B cells, and CD33^+^ myeloid cells in patients with active SLE compared to healthy participants. In this study, peripheral blood mononuclear cells (PBMCs), CD19^+^ B, and CD4^+^ T cells were isolated from active SLE patients and healthy participants. PCR was performed to quantify MALAT1, OAS2, OAS3, and OASL expression in immune cells. MALAT1, OAS2, OAS3, and OASL were knocked down in CD4^+^ T cells to investigate the regulatory effect of MALAT1 on the effectors and their involvement in type I IFNs-mediated inflammation. Results showed higher OAS2, OAS3, and OASL expression in active SLE patients. MALAT1 expression was positively correlated to OAS2, OAS3, and OASL expression in CD19^+^ B or CD4^+^ T cells. MALAT1 knockdown decreased OAS2, OAS3, and OASL expression. Treatment with IFN-α-2a increased the expression of TNF-α, IL-1β, and IFN-α in CD4^+^ T cells. However, knockdown of MALAT1, OAS2, OAS3, and OASL alone inhibited the effect of IFN-α-2a on TNF-α and IL-1β. This study suggested the involvement of MALAT1 in type I IFNs-mediated SLE by up-regulating OAS2, OAS3, and OASL.

## Introduction

Systemic lupus erythematosus (SLE) is an autoimmune disease and its prevalence varies from approximately 20 to 70 per 100,000 people per year, and incidence rates for women are approximately ten times higher than those for men worldwide (1,2). More than 80% of patients with SLE present clinical manifestations such as nephritis, multisystem organ failure, or central nervous system disease. There is a growing understanding of SLE pathogenesis, which is probably associated to an aberrant activation of immune cells and generation of autoantibodies that might induce damage to body tissues and organs (1,2).

Interferons (IFNs) are a group of cytokines with the ability to interfere with and suppress viral replication. There are three types of interferons: type I IFNs, such as IFN-α and IFN-β, type II IFN (e.g., IFN-γ), and type III IFN (e.g., IFN-λ). Of note, type I IFNs have been regarded as central cytokines promoting the occurrence or development of SLE (3,4). Since approximately 1979, it has been known that type I IFN is increased in the sera of SLE patients (5). The role of type I IFN in SLE is not the interference of viral replication but the stimulation of the downstream signaling pathways to promote monocyte differentiation into dendritic cells, block the immune repressive function of Treg cells, support the generation of lymph node-resident follicular helper T cells, and enhance the primary antibody responses of B cells (6). These type I IFN functions lead to abnormal activation of inflammatory cells, more likely resulting in an autoimmune response.

Long non-coding RNAs (lncRNAs) are a novel class of RNAs with a length of more than 200 nucleotides. Although lncRNAs have no protein-encoding capacity, they are involved in the regulation of multiple biological functions via modulating gene expression (7). However, the aberrant expression of lncRNAs make them part of various pathological processes; for example, up-regulated MALAT1 was observed in the immune cells of patients with SLE (8). MALAT1 is an important inflammatory regulator because it can act as a competing endogenous RNA (ceRNA) to interfere with the inhibitory effect of miRNAs on the expression of inflammatory factors (8). Therefore, MALAT1 was proposed to be implied in SLE pathogenesis.

This study initially performed a bioinformatics analysis of data in the GSE10325 data set (https://www.ncbi.nlm.nih.gov/geo/query/acc.cgi?acc=GSE10325) that documented gene expression in CD4^+^ T cells, CD19^+^ B cells, and CD33^+^ myeloid cells in patients with SLE and healthy participants. This analysis revealed the up-regulation of OAS2, OAS3, and OASL (OAS-like) in SLE patients and since OAS2, OAS3, and OASL mediate the pro-inflammatory functions of type I IFN, they are probably involved in SLE pathogenesis. Moreover, bioinformatics analysis uncovered a potential regulatory effect of MALAT1 on OAS2, OAS3, and OASL. Therefore, this study aimed at confirming whether the down-regulation of OAS2, OAS3, and OASL could inhibit the response of T cells to type I IFN through regulating MALAT1.

## Material and Methods

### Subjects

All 17 SLE patients were voluntarily enrolled in this study and were admitted to the Dermatology Department at the First Hospital of Changsha City between June 2018 and January 2019. All of the patients with SLE fulfilled the Systemic Lupus International Collaborating Clinics 2012 criteria (9). Moreover, all included patients had not received any immune modulator or hormonal therapy and immune suppressant drugs. Those patients having acute/chronic infection and malignant tumors were excluded. Their clinical characteristics are reported in Table 1. In China, inactive SLE patients seldom go to hospital to receive systemic therapy. They generally take some immune suppressant drugs and other drugs at home. Therefore, the patients recruited in our hospital in this study presented active SLE.

**Table 1:** Clinical and laboratory characteristics of patients with active systemic lupus erythematosus (SLE).

Characteristics	Patients with SLE (n=17)
No. of males/females	2/15
Age (years)	32 (22-51)
C3 (mg/dL)	26.3±9.7
C4 (mg/dL)	14.27±6.1
Ant-dsDNA antibody (%)	63.2±19
SLEDAI score	9.0±2.4
Red cell count (10^12^/mL)	3.34 (2.31-5.63)
Lymphocyte count (10^9^/mL)	1.34 (0.52-2.8)
Platelet count (10^9^/mL)	161 (85-359)
Serum interleukin-1 (pg/mL)	1.13 (0.76-1.34)
Tumor necrosis factor-α (pg/mL)	9.05 (6.88-1.16)

Data are reported as means±SD or median (range). SLEDAI: SLE Disease Activity Index.

SLE Disease Activity Index (SLEDAI) score >6 was used to discriminate active SLE patients from quiescent ones. Clinical manifestations of patients with active SLE are shown in Table 2. In addition, 25 age- and sex-matched healthy controls with no arthralgia, heart failure, renal failure, or autoimmune disease, and free from other inflammatory conditions, were recruited for this study. All subjects who participated in the research provided written informed consent. The research protocol was approved by the Medical Ethical Committee of the hospital.

**Table 2:** Clinical manifestations of patients with active systemic lupus erythematosus (n=17).

Clinical manifestations	Number of patients
Malar rash/photosensitive rash/acute cutaneous lupus	17
Renal disorder	9
Urine protein (>0.5 g/24 h or +++)	9
WBC/HP>5^a^	7
RBC/HP>5^a^	6
Neurological symptoms	12
Headache	12
Epilepsy	2
Arthritis	6
Oral ulcers	8
Non-scarring alopecia	15

WBC: white blood cell; HP: high power lens; RBC: red blood cell. ^a^Patients did not have kidney stones or infection.

### Isolation of PBMC, CD19^+^ B, and CD4^+^ T cells from blood

Peripheral blood mononuclear cells (PBMCs) were isolated from the blood of SLE patients and healthy participants using human peripheral blood lymphocyte separation medium (Solarbio, China) through a Ficoll-Hypaque density-gradient centrifugation method (444 *g*, 20 min, 27°C). CD19^+^ B and CD4^+^ T cells were further purified from PBMCs using specific magnetic beads (Solarbio) and the purity was evaluated by flow cytometry (purity >90%, Becton Dickinson, USA).

### Cell culture and treatment

The isolated CD4+ T cells were cultured in RPMI 1640 medium containing 10% FBS and 1% penicillin/streptomycin at 37°C in a humidified atmosphere containing 5% CO_2_ (MCO-175, Sanyo, Japan). For transfection, CD4+ T cells were seeded into 24-well plates at a density of 2×10^4^ cells/well and then transfected with siRNAs specific to MALAT1, OAS2, OAS3, and OASL using Lipofectamine^TM^ RNAiMAX (Invitrogen, USA) according to the manufacturer's instructions. After 12 h, the efficiency of siRNAs-mediated knockdown was evaluated using PCR. Human IFN-α-2a recombinant protein (1000 units/mL, Thermo Fisher Scientific, USA) was added to CD4^+^ T cells. Cell viability was assessed at 12 and 24 h after the IFN-α-2a treatment.

### Cell viability assay

Cell Counting Kit-8 (CCK-8, Dojindo Molecular Technologies, China) assay was used to assess cell viability. Briefly, CD4+ T cells were seeded into 96-well plates with 5×10^3^ cells/well. After the above-mentioned treatments, 10 μL of CCK-8 solution was added into the culture medium of each well and the plates were incubated for another 1 h at 37°C. Then, the absorbance (450 nm) of each well was measured with a microplate reader (Bio-Tek Instruments, USA).

### Real-time quantitative PCR (RT-qPCR)

Total RNA was extracted from CD4^+^ T cells using Trizol reagent (Life Technologies Corporation, USA) according to the manufacturer's instructions. The extracted RNA was reversed using the Multiscribe™ Reverse Transcription Kit (Applied Biosystems, USA). qPCR was performed with the One Step SYBR^®^ PrimeScript^®^ PLUS RT-PCR kit (TaKaRa Biotechnology, China) on the ABI PRISM 7500 real-time PCR System (Applied Biosystems). All primers were synthesized by GenePharma (China) and the primer sequences are reported in Table 3. Results were calculated using the classic 2^-△△Ct^ method and normalized to that of glyceraldehyde-3-phosphate dehydrogenase (GAPDH).

**Table 3:** Primers used for PCR assay.

Gene name	Direction	Sequence (5′-3′)	Tm	Amplification Size
OAS2	Forward	CTCAGAAGCTGGGTTGGTTTAT	60	76
	Reverse	ACCATCTCGTCGATCAGTGTC		
OAS3	Forward	GAAGGAGTTCGTAGAGAAGGCG	62	114
	Reverse	CCCTTGACAGTTTTCAGCACC		
OASL	Forward	CTGATGCAGGAACTGTATAGCAC	61	105
	Reverse	CACAGCGTCTAGCACCTCTT		
TNF-α	Forward	CCTCTCTCTAATCAGCCCTCTG	60	220
	Reverse	GAGGACCTGGGAGTAGATGAG		
IL-1β	Forward	ATGATGGCTTATTACAGTGGCAA	60	132
	Reverse	GTCGGAGATTCGTAGCTGGA		
IFN-α	Forward	GCCTCGCCCTTTGCTTTACT	62	89
	Reverse	CTGTGGGTCTCAGGGAGATCA		
MALAT1	Forward	CAGTGGGGAACTCTGACTCG	61	263
	Reverse	GTGCCTGGTGCTCTCTTACC		
GAPDH	Forward	GGAGCGAGATCCCTCCAAAAT	61	197
	Reverse	GGCTGTTGTCATACTTCTCATGG		

### Western blotting

Protein samples used for western blotting were extracted using RIPA lysis buffer (Beyotime Biotechnology, China) supplemented with protease inhibitors (Roche, China). The protein samples were quantified by the BCA™ Protein Assay Kit (Beyotime Biotechnology). Then, equal amounts of samples were separated by 10–12% sodium dodecyl sulphate polyacrylamide gel electrophoresis gels and transferred onto polyvinylidene difluoride membranes (Millipore, USA). The membranes were incubated overnight at 4°C with primary antibodies p-Jak1 (1:500; ab138005; Abcam Biotechnology, USA), p-Stat3 (1:500; ab30647; Abcam), and GAPDH (1:1000; ab8245; Abcam). Then, membranes were washed and incubated with horseradish peroxidase-marked secondary antibodies for 1 h at room temperature. After that, membranes were transferred into the Bio-Rad ChemiDoc™ XRS system (Bio-Rad Laboratories, USA) and 200 μL of Immobilon Western Chemiluminescent HRP Substrate (Millipore) was added to cover the membrane surface. Protein signals were captured and the intensities of the bands were quantified with the Bio-Rad Image™ 3.0 version software (Bio-Rad).

### Statistical analysis

Data are reported as means±SD. All analyses were performed using the SPSS 19.0 software (IBM, USA). Student's *t*-test was used for comparison between two groups. One-way ANOVA followed by Bonferroni test was used for comparison among 3 or more groups. Correlations were analyzed using the Pearson's correlation coefficient. P<0.05 was considered to be statistically significant.

## Results

### Implication of OAS2, OAS3, and OASL in SLE pathogenesis

**Figure 1:**
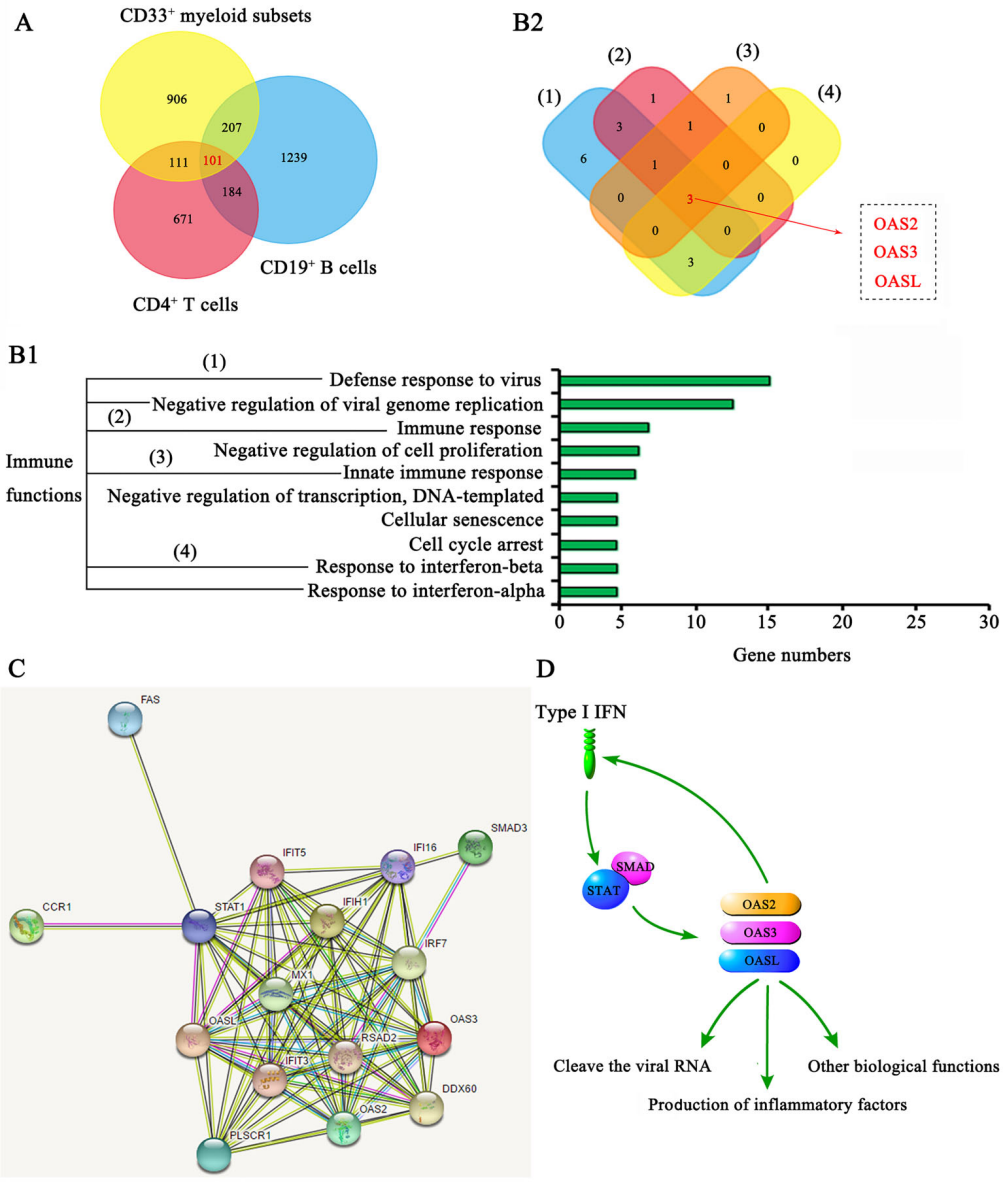
Bioinformatics analysis revealed the implication of OAS2, OAS3, and OASL in systemic lupus erythematosus (SLE) pathogenesis. **A**, The GSE10325 data set recorded the expression of genes in CD4^+^ T cells, CD19^+^ B cells, and CD33^+^ myeloid cells in SLE patients and healthy participants. The Venn diagram shows that 101 genes in all CD4^+^ T cells, CD19^+^ B cells, and CD33^+^ myeloid cells were expressed differently between SLE patients and healthy participants. **B1**, A GO enrichment analysis (https://david.ncifcrf.gov/) was performed to reveal biological processes affected by these 101 genes. **B2**, The Venn diagram shows that three genes (OAS2, OAS3, and OASL) affected all the immunity-related functions including defense response to virus, immune response, innate immunity, and response to interferon. **C**, Protein-protein interactions among immune-related genes. **D**, Schematic diagram showing that type I interferon (IFN) increased the expression and functions of OAS2, OAS3, and OASL through STATS and SMAD signals. In addition to the interference of viral replication, OAS2, OAS3, and OASL also promoted the production of inflammatory factors such as type I IFN.

The GSE10325 data set recorded the expression of genes in CD4^+^ T cells, CD19^+^ B cells, and CD33^+^ myeloid cells in SLE patients and healthy participants. The Venn diagram showed that 101 genes in all CD4^+^ T cells, CD19^+^ B cells, and CD33^+^ myeloid cells expressed differently between SLE patients and healthy participants (Figure 1A). A GO enrichment analysis (https://david.ncifcrf.gov/) was performed to reveal biological processes affected by these 101 genes. These genes dramatically affected immunity-related functions including defense response to virus, immune response, innate immune response, and response to IFN (Figure 1B1). The Venn diagram revealed that three genes (OAS2, OAS3, and OASL) affected all these functions (Figure 1B2). We analyzed the interaction among immune-related genes among the 101 genes (Figure 1C). Results suggested that Fas, SMAD, and CCR1 signals were probably involved in the regulation of these immune-related genes; and OAS2, OAS3, and OASL played important roles in the response of immune cells to type I IFN. As shown in the schematic diagram (Figure 1D), type I IFN increased the expression and functions of OAS2, OAS3, and OASL through STATS and SMAD signals. In addition to the interference of viral replication, OAS2, OAS3, and OASL also promoted the production of inflammatory factors such as type I IFN.

### OAS2, OAS3, and OASL were up-regulated in immune cells in active SLE patients

**Figure 2:**
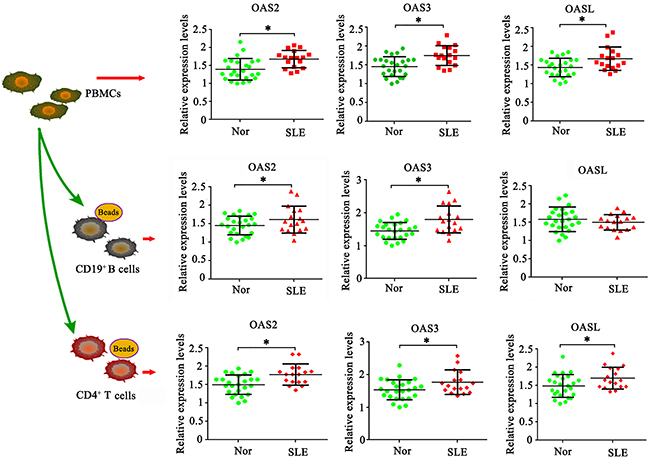
OAS2, OAS3, and OASL were up-regulated in immune cells in systemic lupus erythematosus (SLE) patients. PCR assay was performed to evaluate OAS2, OAS3, and OASL expression in peripheral blood mononuclear cells (PBMCs), CD19^+^ B cells, and CD4^+^ T cells in patients with SLE and healthy participants (Nor). Horizontal lines indicate median and interquartile range. *P<0.05 *vs* Nor (Student’s *t*-test).

To confirm the data of OAS2, OAS3, and OASL from the GSE10325 data set, we performed a PCR assay in PBMCs, CD19^+^ B, and CD4^+^ T cells in patients with active SLE and healthy participants. OAS2, OAS3, and OASL were up-regulated in PBMCs in active SLE patients compared to healthy participants (P<0.05, Figure 2). OASL expression in CD19^+^ B cells showed no significant difference between active SLE patients and healthy participants, but OAS2 and OAS3 expression in CD19^+^ B were notably up-regulated in active SLE patients (P<0.05). CD4^+^ T cells from active SLE patients showed increased expression of all OAS2, OAS3, and OASL compared to healthy participants (P<0.05).

Active SLE patients show various clinical manifestations such as cutaneous and oral lesions, renal disorders, arthritis, and neurological symptoms. This study analyzed the OAS2, OAS3, and OASL expression in patients with or without these clinical manifestations. Active SLE patients with renal disorders showed higher OAS2 and OASL expression in PBMCs, OAS3 and OASL expression in CD19^+^ B cells, and OAS3 expression in CD4^+^ T cells than patients without the symptoms (P<0.05, Tables 4, 5, and 6). Active SLE patients with arthritis showed higher OAS2 and OAS3 expression in PBMCs and CD19^+^ B cells and OAS2 expression in CD4^+^ T cells (P<0.05, Tables 4, 5, and 6).

**Table 4:** Association of OAS2, OAS3, and OASL expression in peripheral blood mononuclear cells with systemic lupus erythematosus manifestations.

Clinical manifestations	Expression level	Expression level	P value
Malar rash/photosensitive rash/acute cutaneous lupus	Yes (n=17)	No (n=0)	
OAS2	1.64±0.22		-
OAS3	1.75±0.26		-
OASL	1.72±0.25		-
Renal disorder	Yes (n=9)	No (n=8)	
OAS2	1.73±0.19	1.55±0.15	0.03
OAS3	1.79±0.38	1.71±0.3	0.054
OASL	1.81±0.27	1.63±0.23	0.039
Neurological symptoms	Yes (n=12)	No (n=5)	
OAS2	1.61±0.35	1.67±0.37	0.151
OAS3	1.71±0.38	1.79±0.28	0.238
OASL	1.65±0.34	1.79±0.38	0.117
Arthritis	Yes (n=6)	No (n=11)	
OAS2	1.69±0.24	1.59±0.21	0.047
OAS3	1.83±0.25	1.67±0.28	0.033
OASL	1.78±0.37	1.66±0.22	0.069
Oral ulcers	Yes (n=11)	No (n=6)	
OAS2	1.6±0.25	1.68±0.31	0.078
OAS3	1.81±0.33	1.69±0.27	0.061
OASL	1.77±0.38	1.67±0.4	0.095
Non-scarring alopecia	Yes (n=15)	No (n=2)	
OAS2	1.75±0.41	1.53±0.25	-
OAS3	1.71±0.35	1.79±0.21	-
OASL	1.66±0.37	1.78±0.35	-

Data are reported as means±SD. Groups were compared with Student’s *t*-test.

**Table 5:** Association of OAS2, OAS3, and OASL expression in CD19^+^ B cells with systemic lupus erythematosus manifestations.

Clinical manifestations	Expression level	Expression level	P value
Malar rash/photosensitive rash/acute cutaneous lupus	Yes (n=17)	No (n=0)	
OAS2	1.73±0.57		-
OAS3	1.79±0.61		-
OASL	1.48±0.26		-
Renal disorder	Yes (n=9)	No (n=8)	
OAS2	1.88±0.42	1.58±0.38	0.057
OAS3	1.99±0.46	1.59±0.33	0.042
OASL	1.63±0.28	1.33±0.21	0.038
Neurological symptoms	Yes (n=12)	No (n=5)	
OAS2	1.71±0.55	1.75±0.48	0.211
OAS3	1.8±0.51	1.78±0.37	0.153
OASL	1.57±0.31	1.39±0.4	0.097
Arthritis	Yes (n=6)	No (n=11)	
OAS2	1.91±0.41	1.55±0.35	0.021
OAS3	1.88±0.46	1.7±0.44	0.039
OASL	1.68±0.37	1.28±0.35	0.054
Oral ulcers	Yes (n=11)	No (n=6)	
OAS2	1.79±0.48	1.67±0.46	0.089
OAS3	1.87±0.55	1.71±0.36	0.076
OASL	1.57±0.38	1.39±0.31	0.107
Non-scarring alopecia	Yes (n=15)	No (n=2)	
OAS2	1.85±0.59	1.61±0.28	-
OAS3	1.91±0.58	1.67±0.31	-
OASL	1.56±0.32	1.4±0.21	-

Data are reported as means±SD. Groups were compared with Student’s *t*-test.

**Table 6:** Association of OAS2, OAS3, and OASL expression in CD4^+^ T cells with systemic lupus erythematosus manifestations.

Clinical manifestations	Expression level	Expression level	P value
Malar rash/photosensitive rash/acute cutaneous lupus	Yes (n=17)	No (n=0)	
OAS2	1.75±0.49		-
OAS3	1.73±0.51		-
OASL	1.69±0.24		-
Renal disorder	Yes (n=9)	No (n=8)	
OAS2	1.83±0.47	1.67±0.42	0.089
OAS3	1.9±0.46	1.56±0.39	0.045
OASL	1.78±0.37	1.6±0.25	0.072
Neurological symptoms	Yes (n=12)	No (n=5)	
OAS2	1.79±0.35	1.71±0.31	0.231
OAS3	1.86±0.41	1.6±0.35	0.128
OASL	1.67±0.37	1.71±0.4	0.177
Arthritis	Yes (n=6)	No (n=11)	
OAS2	1.98±0.41	1.52±0.38	0.048
OAS3	1.88±0.45	1.58±0.34	0.073
OASL	1.79±0.39	1.59±0.28	0.089
Oral ulcers	Yes (n=11)	No (n=6)	
OAS2	1.82±0.42	1.68±0.36	0.088
OAS3	1.77±0.38	1.69±0.27	0.091
OASL	1.53±0.35	1.85±0.51	0.059
Non-scarring alopecia	Yes (n=15)	No (n=2)	
OAS2	1.79±0.45	1.71±0.28	-
OAS3	1.81±0.5	1.65±0.33	-
OASL	1.66±0.31	1.72±0.26	-

Data are reported as means±SD. Groups were compared with Student’s *t*-test.

### MALAT1 was involved in the regulation of OAS2, OAS3, and OASL in immune cells

**Figure 3:**
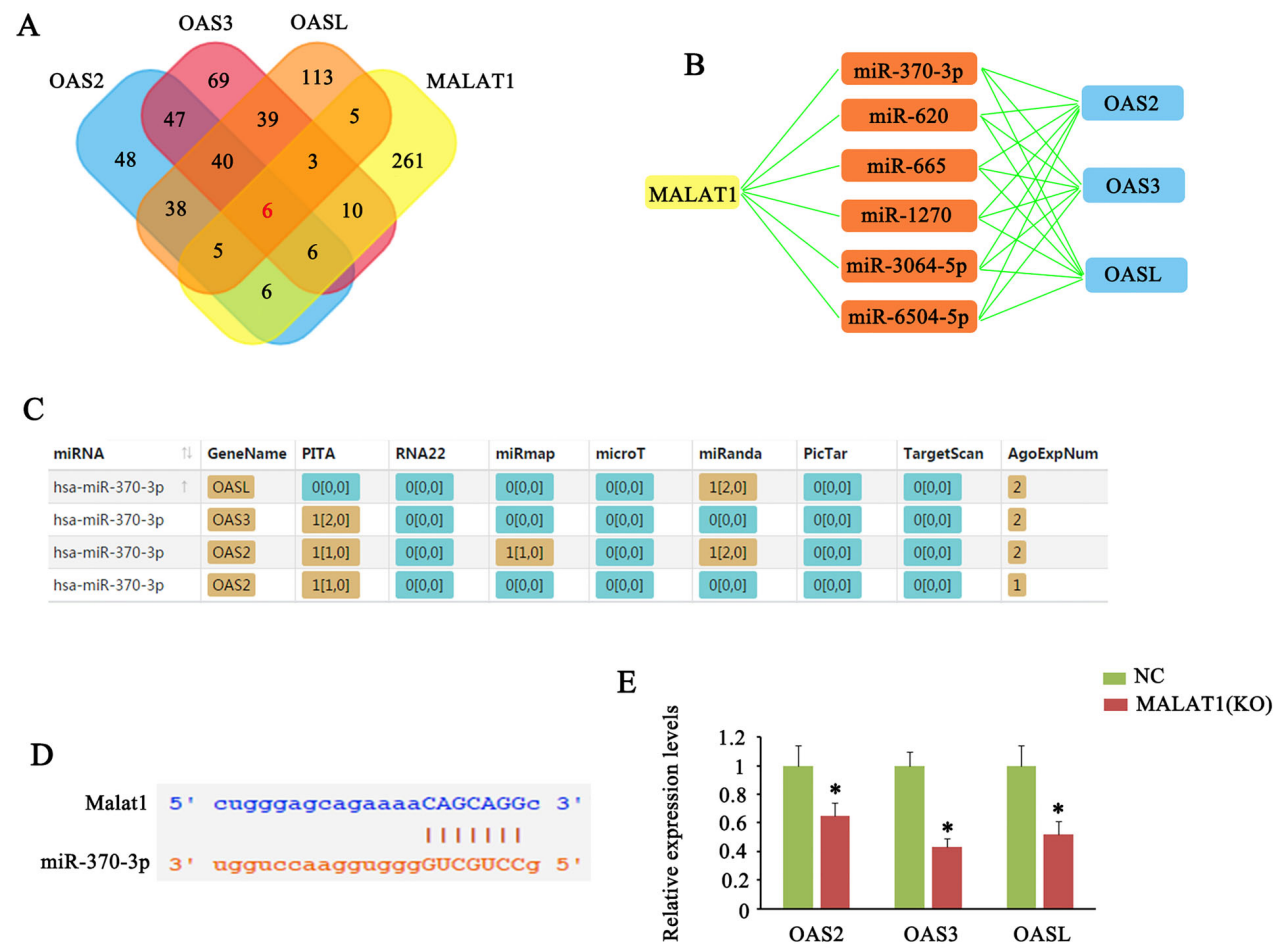
MALAT1 had potential effects on OAS2, OAS3, and OASL expression by interacting with miRNAs. **A**, The Venn diagram shows that six miRNAs can target all OAS2, OAS3, OASL, and MALAT1. **B**, MALAT1 can function as a ceRNA of these six miRNAs to block their effects on OAS2, OAS3, and OASL mRNA. **C,** For example, miR-370-3p was predicted to target OAS2, OAS3, and OASL. **D**, At the same time, miR-370-3p could also bind to MALAT1. **E**, PCR analysis showed that OAS2, OAS3, and OASL expression was also decreased with the knockdown of MALAT1. Data are reported as means±SD. *P<0.05 *vs* NC (negative control) (Student’s *t*-test). KO: knocked out.

This study used various softwares for analysis, including PITA, RNA22, miRmap, mircoT, miRanda, PicTar, and Targetscan to find miRNAs targeting OAS2, OAS3, OASL, and MALAT1. The Venn diagram showed that six miRNAs can target all OAS2, OAS3, OASL, and MALAT1 (Figure 3A). It is possible that MALAT1 can function as a ceRNA of these six miRNAs to block their effects on OAS2, OAS3, and OASL (Figure 3B). For example, miR-370-3p was predicted to target OAS2, OAS3, and OASL (Figure 3C), while miR-370-3p could also bind to MALAT1 (Figure 3D).

**Figure 4:**
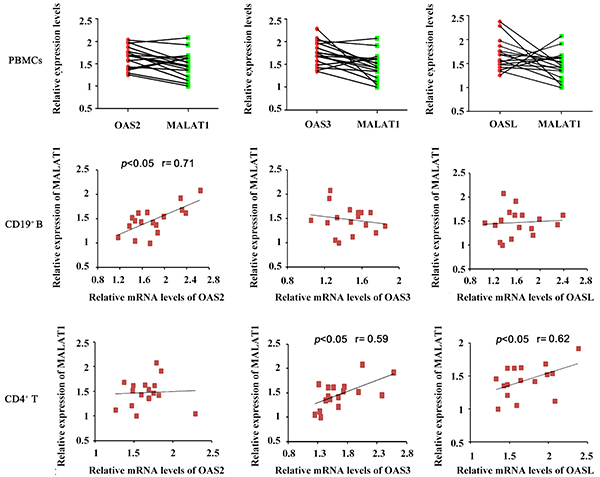
Positive correlation of MALAT1 expression with OAS2, OAS3, and OASL expression in immune cells. Pearson's correlation analysis was used to determine the correlation of MALAT1 expression with OAS2, OAS3, and OASL expression in peripheral blood mononuclear cells (PBMCs), CD19^+^ B cells, and CD4^+^ T cells from systemic lupus erythematosus patients.

To confirm the regulatory effect of MALAT1 on OAS2, OAS3, and OASL, we knocked down MALAT1 in CD4^+^ T cells. PCR analysis showed that OAS2, OAS3, and OASL expression was also decreased with knockdown (P<0.05, Figure 3E). We also evaluated the correlation of MALAT1 with OAS2, OAS3, and OASL in their expression in PBMCs cells, CD19^+^ B cells, and CD4^+^ T cells from SLE patients. OAS2, OAS3, and OASL expression had no correlation with that of MALAT1 in PBMCs cells (Figure 4). However, OAS2 expression in CD19^+^ B cells as well as OAS3 and OASL expression in CD4^+^ T cells were positively correlated with MALAT1 expression in these cells (P<0.05).

### Down-regulation of MALAT1 inhibited the response of T cells to type I IFN by down-regulating OAS2, OAS3, and OASL

**Figure 5:**
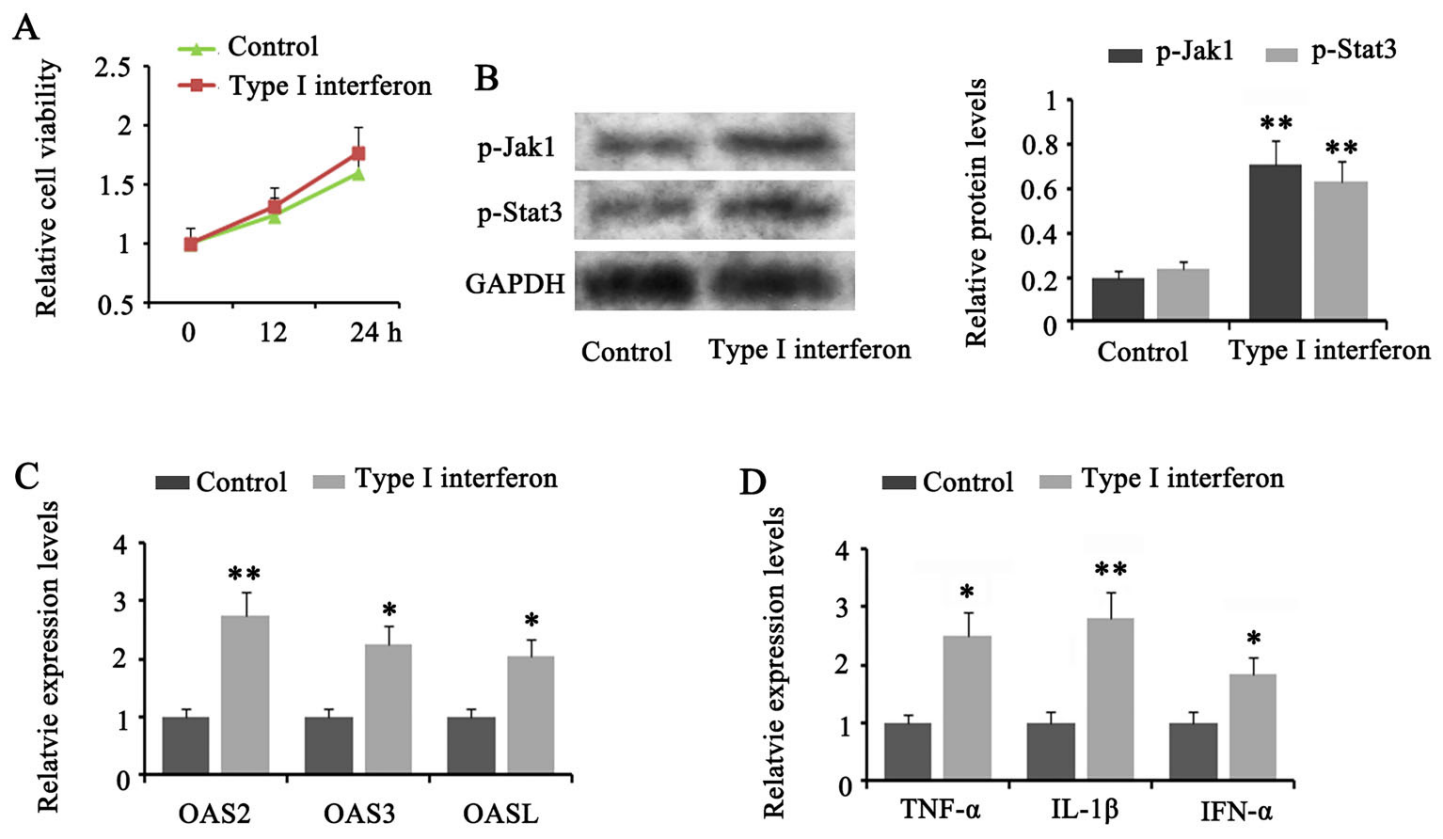
Type I interferon (IFN) increased expression of OAS2, OAS3, OASL, and inflammatory factors in T cells. CD4+ T cells were treated with IFN-α-2a. **A**, IFN-α-2a had no significant effect on the cell viability within 24 h. **B**, Western blot was performed to evaluate phosphorylation levels of Jak1 and Stat3. **C**, PCR was performed to evaluate OAS2, OAS3, and OASL expression in T cells. **D**, PCR was performed to evaluate expression of inflammatory factors in T cells. Data are reported as means±SD. *P<0.05, **P<0.01 *vs* control (Student’s *t*-test).

Treating CD4^+^ T cells with IFN-α-2a had no significant effect on cell viability within 24 h (Figure 5A). However, phosphorylation levels of Jak1 (P<0.01) and Stat3 (P<0.01) were increased by IFN-α-2a treatment (Figure 5B). IFN-α-2a treatment increased OAS2 (P<0.01), OAS3 (P<0.05), and OASL (P<0.05) expression levels in CD4^+^ T cells (Figure 5C). Moreover, the expression of TNF-α (P<0.05), IL-1β (P<0.01), and IFN-α (P<0.05) in CD4^+^ T cells was also increased by IFN-α-2a (Figure 5D).

**Figure 6:**
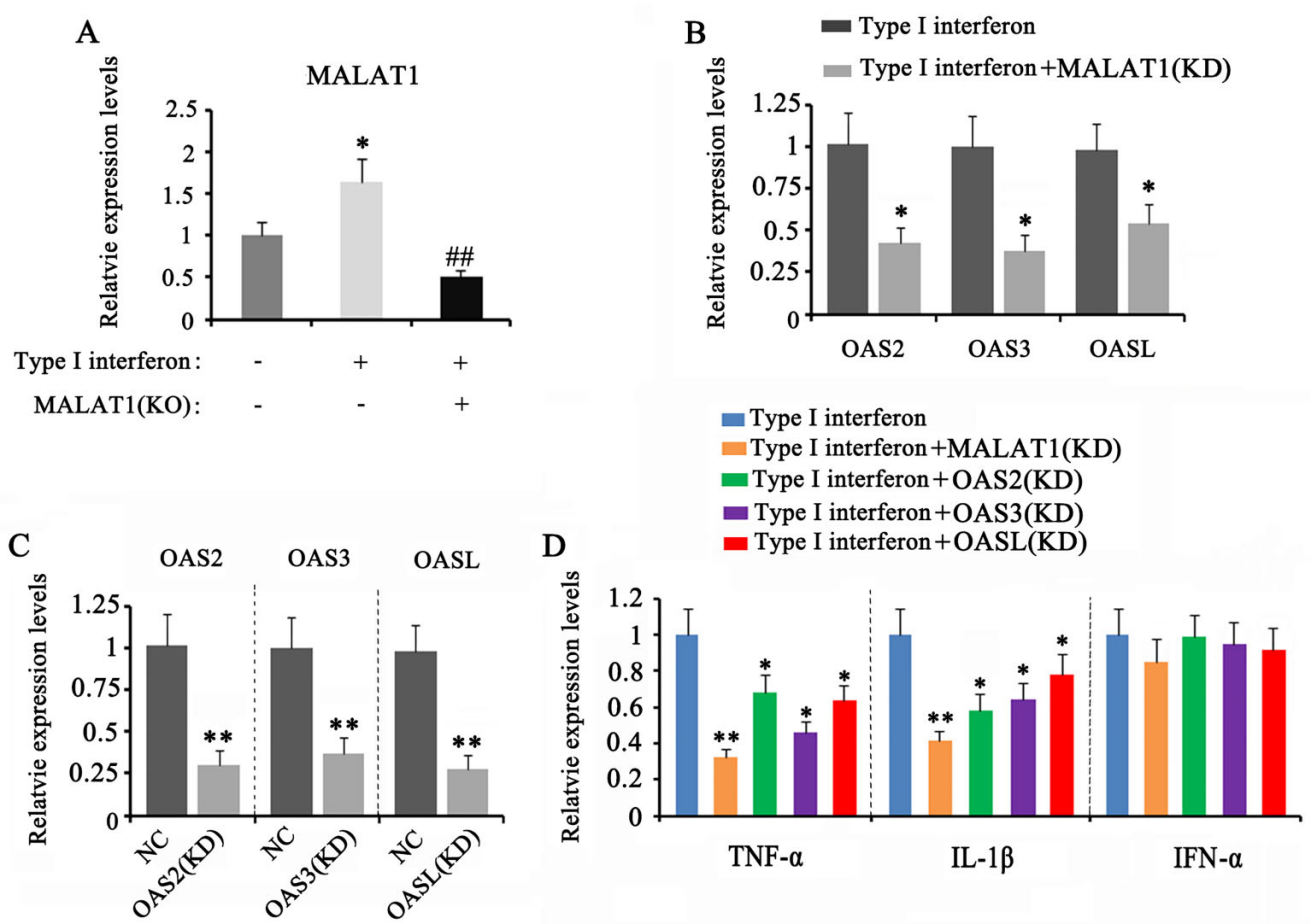
Down-regulation of MALAT1 inhibited the response of T cells to type I interferon (IFN) by down-regulating OAS2, OAS3, and OASL. CD4+ T cells were treated with IFN-α-2a for 24 h and analyzed by PCR. **A**, MALAT1 expression after transfection with siRNA-MALAT1. **B**, OAS2, OAS3, and OASL expression after transfection with siRNA-MALAT1. **C**, OAS2, OAS3, and OASL expression after transfection with their siRNAs. **D**, Inflammatory factors after silencing OAS2, OAS3, OASL, and MALAT1. Data are reported as means±SD. *P<0.05, **P<0.01 *vs* control (NC); # #P<0.01 *vs* IFN (ANOVA). KO: knocked out; KC: knocked down.

Treatment with IFN-α-2a increased MALAT1 expression in CD4^+^ T cells, but transfection with siRNA-MALAT1 before IFN-α-2a treatment conversely down-regulated MALAT1 expression (P<0.01 *vs* IFN-α-2a treatment group, Figure 6A). The down-regulation of MALAT1 attenuated the increase of OAS2 (P<0.05), OAS3 (P<0.05), and OASL (P<0.05) expression caused by IFN-α-2a (Figure 6B). To determine the role of OAS2, OAS3, and OASL in IFN-α-2a-mediated inflammation, we silenced them alone in CD4^+^ T cells (P<0.01, Figure 6C). Depletion of MALAT1, OAS2, OAS3, and OASL decreased TNF-α (P<0.05, P<0.01) and IL-1β (P<0.05, P<0.01) expression in CD4^+^ T cells upon IFN-α-2a treatment, while it had no effect on IFNα expression (Figure 6D).

## Discussion

SLE is associated with an aberrant overactivation of immune functions that results in tissue and organ damage in the whole body (1,2). However, the molecular mechanisms underlying the overactivation of immune functions remain not fully understood. According to data in the GSE10325 data set, many immune-related genes were aberrantly expressed in CD4^+^ T cells, CD19^+^ B cells, and CD33^+^ myeloid cells in SLE patients. Yet, OAS2, OAS3, and OASL may be tightly linked to SLE pathogenesis among these immune-related genes for the following reasons: 1) they are up-regulated in all CD4^+^ T cells, CD19^+^ B cells, and CD33^+^ myeloid cells in SLE patients and 2) they participate in various immune functions, especially considering the response to IFN. OAS2, OAS3, and OASL belong to the 2′-5′-oligoadenylate synthetase (OAS) family. Expression of OAS-family genes is induced by stimulation of type I and II IFNs (10,11). In the case of a viral infection, OAS family proteins polymerize ATP to 2′-5′-linked adenosine oligomers that can activate the RNase L degradative pathway to cleave the viral RNA and control the infection (12,13). However, apart from the anti-virus function, OAS family proteins may be also related to SLE pathogenesis. Grammatikos et al. suggested that combining the expression levels of OAS2, CD70, and IL10 in T cells can be used for diagnosis and monitoring of disease activity in SLE patients (14–16). A “T-cell score”, which was created with the three gene panel, was significantly higher in SLE patients than in the healthy control group and correlated significantly with dsDNA, PGA, SLEDAI, and ESR levels (14). Braunstein et al. (15) found that an IFN score, which was determined by OASL, OAS1, LY6E, ISG15, and MX1 expression, was higher in patients with subacute cutaneous lupus erythematosus and discoid lupus erythematosus than in healthy controls. Moreover, the IFN score was positively correlated with the Cutaneous Lupus Area and Severity Index (15). OAS3 expression in B cells was also used as a marker for SLE diagnosis, especially in male patients suspected to have SLE (16).

This study confirmed the up-regulation of OAS2, OAS3, and OASL in PBMCs cells and CD4^+^ T cells as well as up-regulation of OAS3 and OASL in CD19^+^ B cells in active SLE patients. Moreover, active SLE patients with renal disorders and arthritis showed higher OAS2, OAS3, and OASL expression than patients without these symptoms. These data suggested the implication of OAS2, OAS3, and OASL in the pathogenesis of active SLE, especially for patients with renal disorders and arthritis symptoms. In this study, the expression of OAS2, OAS3, and OASL in these immune cells was not measured in quiescent SLE patients, which is a limitation. Further study is planned to determine whether there is difference in their expression between quiescent SLE patients and healthy controls.

Type I IFN includes 13 genes, including IFNα, IFNβ, and other less explored members (17 [Bibr B18]). A number of studies have reported the association between high mRNA expression and serum levels of IFNα and activity of SLE disease, suggesting that IFNα is an important signature of SLE disease (17–19). This study treated CD4^+^ T cells with IFN-α-2a, which resulted in the increase of OAS2, OAS3, and OASL expression. Previous studies showed significant association between OAS2, OAS3, and OASL expression and IFNα level during infection of virus or bacteria (20,21). OAS2, OAS3, and OASL were further involved in the production of TNF-α, IL-1β, and IFNs in the presence of virus or bacteria (12,22). However, many SLE patients suffer neither from a viral nor a bacterial infection. Therefore, it is necessary to determine whether OAS2, OAS3, and OASL are still involved in the production of TNF-α, IL-1β, and IFNs in the absence of virus and bacteria. In the present study, treatment with IFN-α-2a increased phosphorylation levels of Jak1 and Stat3, suggesting the activation of downstream signal of type I IFN (23,24). IFN-α-2a treatment was associated with the increase of the expression of TNF-α, IL-1β, and IFN-α. However, knockdown of OAS2, OAS3, and OASL to some extent inhibited the increase of TNF-α and IL-1β. These data suggested that OAS2, OAS3, and OASL mediated the pro-inflammatory effects of IFN-α-2a in the absence of virus and bacteria. Therefore, this study revealed the pathogenic mechanism of OAS2, OAS3, and OASL in SLE patients.

Considering the important roles of OAS2, OAS3, and OASL in SLE pathogenesis, it is necessary to find an approach to down-regulate these effectors in immune cells. Up-regulated MALAT1 was also observed in immune cells in SLE patients (8). This study found a correlation of MALAT1 expression with OAS2, OAS3, and OASL in some immune cells. Bioinformatics analysis showed that MALAT1 can competitively bind to six miRNAs with mRNAs of OAS2, OAS3, and OASL. The analysis implied that MALAT1 was able to disrupt the miRNA-mediated degradation of OAS2, OAS3, and OASL mRNAs, thus increasing their expression in turn. To test this hypothesis, we knocked down MALAT1 in CD4^+^ T cells. OAS2, OAS3, and OASL expression was also down-regulated after MALAT1 knockdown. MALAT1 knockdown also blocked the production of TNF-α and IL-1β in response to IFN-α-2a. Therefore, MALAT1 might be an important therapeutic target for SLE because of its promoting effect on OAS2, OAS3, and OASL. There is no evidence indicating a direct association between MALAT1 and type I IFN. However, MALAT1 might regulate signaling pathway downstream of type I IFN, because a study showed that silencing MALAT1 attenuated the activation of the JAK/STAT pathway in a tumor cell (25). Further study is needed to investigate the correlation between MALAT1 and type I IFN in SLE patients and the interaction between MALAT1 and type I IFN in immune cells.

In summary, this study found that OAS2, OAS3, and OASL mediated the pro-inflammatory effects of IFN-α-2a in the absence of virus and bacteria. This is probably an important pathogenic mechanism of OAS2, OAS3, and OASL in active SLE. Silencing MALAT1 effectively blocks the increase of OAS2, OAS3, and OASL in T cells in response to IFN-α-2a suggesting that this might be a valid approach to control OAS2, OAS3, and OASL expression in the cells.
